# Prescriptions for Buprenorphine in Michigan Following an Education Intervention

**DOI:** 10.1001/jamanetworkopen.2023.49103

**Published:** 2023-12-21

**Authors:** Liying Chen, Sheba Sethi, Cara Poland, Christopher Frank, Elizabeth Tengelitsch, Jason Goldstick, Jeremy B. Sussman, Amy S. B. Bohnert, Lewei (Allison) Lin

**Affiliations:** 1 Department of Biostatistics, University of Michigan, Ann Arbor; 2 Department of Anesthesiology, University of Michigan, Ann Arbor; 3 Department of Obstetrics, Gynecology and Reproductive Health, Michigan State University, East Lansing; 4 Department of Family Medicine, University of Michigan, Ann Arbor; 5 Institute for Health Policy and Innovation, University of Michigan, Ann Arbor; 6 Department of Psychiatry, University of Michigan, Ann Arbor; 7 Injury Prevention Center, University of Michigan, Ann Arbor; 8 Department of Emergency Medicine, University of Michigan, Ann Arbor; 9 Department of Health Behavior and Health Education, University of Michigan, Ann Arbor; 10 Division of General Internal Medicine, University of Michigan, Ann Arbor; 11 Center for Clinical Management Research, VA Ann Arbor Healthcare System, Ann Arbor

## Abstract

**Importance:**

Buprenorphine is an underused treatment for opioid use disorder (OUD) that can be prescribed in general medical settings. Founded in 2017, the Michigan Opioid Collaborative (MOC) is an outreach and educational program that aims to address clinician and community barriers to buprenorphine access; however, the association between the MOC and buprenorphine treatment is unknown.

**Objective:**

To evaluate the association between MOC service use and county-level temporal trends of density of buprenorphine prescribers and patients receiving buprenorphine.

**Design, Setting, and Participants:**

This cohort study exploited staggered implementation of MOC services across all Michigan counties. Difference-in-difference analyses were conducted by applying linear fixed-effects regression across all counties to estimate the overall association of MOC engagement with outcomes and linear regression for each MOC-engaged county separately to infer county-specific results using data from May 2015 to August 2020. Analyses were conducted from September 2021 to November 2023.

**Exposures:**

MOC engagement.

**Main Outcomes and Measures:**

County-level monthly numbers of buprenorphine prescribers and patients receiving buprenorphine (per 100 000 population).

**Results:**

Among 83 total counties, 57 counties (68.7%) in Michigan were engaged by MOC by 2020, with 3 (3.6%) initiating engagement in 2017, 19 (22.9%) in 2018, 27 (32.5%) in 2019, and 8 (9.6%) in 2020. Michigan is made up of 83 counties with a total population size of 9 990 000. A total of 5 070 000 (50.8%) were female, 1 410 000 (14.1%) were African American or Black, 530 000 (5.3%) were Hispanic or Latino, and 7 470 000 (74.7%) were non-Hispanic White. The mean (SD) value of median age across counties was 44.8 (6.4). The monthly increases in buprenorphine prescriber numbers in the preengagement (including all time points for nonengaged counties) and postengagement periods were 0.07 and 0.39 per 100 000 population, respectively, with the absolute difference being 0.33 (95% CI, 0.12-0.53) prescribers per 100 000 population (*P* = .002). The numbers of patients receiving buprenorphine increased by an average of 0.6 and 7.15 per 100 000 population per month in preengagement and postengagement periods, respectively, indicating an estimated additional 6.56 (95% CI, 2.09-11.02) patients receiving buprenorphine per 100 000 population (*P* = .004) monthly increase after engagement compared with before.

**Conclusions and Relevance:**

In this cohort study measuring buprenorphine prescriptions in Michigan over time, counties’ engagement in OUD-focused outreach and clinician education services delivered by a multidisciplinary team was associated with a temporal increase in buprenorphine prescribers and patients receiving buprenorphine.

## Key Points

**Question:**

Can buprenorphine prescribing be increased by the implementation of a statewide, flexible outreach and educational program that addresses the wide range of barriers faced by different communities?

**Findings:**

In this cohort study of 83 Michigan counties, receipt of services from the Michigan Opioid Collaborative was associated with county-level increases in density of buprenorphine prescribers and patients filling prescriptions for buprenorphine.

**Meaning:**

These findings suggest flexible outreach and free clinician and community education delivered by a multidisciplinary team is a scalable model for expanding access to buprenorphine in diverse communities.

## Teaser

This cohort study evaluates the association between Michigan Opioid Collaborative service use and county-level temporal trends of density of buprenorphine prescribers and patients receiving buprenorphine.

## Introduction

Opioid use disorder (OUD) continues to cause significant morbidity and mortality in the US.^1^ The reference standard treatments are medications for OUD (MOUD), including buprenorphine and methadone.^2^ However, most people with OUD do not receive MOUD,^3,4^ even after critical events such as an overdose.^5^ Buprenorphine is particularly poised for widespread expansion, since it can be prescribed in a general medical setting, unlike methadone, which can only be dispensed in regulated treatment programs.

As of 2017, 60% of rural counties in the US lacked a MOUD practitioner,^6^ though there are still treatment gaps in nonrural counties.^7^ Many practitioners with relevant training cite several barriers to prescribing buprenorphine, including insufficient knowledge; time constraints; lack of institutional, peer, and psychosocial support; lack of specialty backup for complex problems; regulatory concerns; and low reimbursement.^6^ Interventions to increase buprenorphine treatment include removing barriers to prescribing,^8^ increasing prescriptive authority for nurse practitioners and physician assistants,^9^ expanding telehealth for MOUD,^3,10,11^ and providing educational outreach.^12^ Although many of these interventions show promise, most only address a subset of the barriers. Furthermore, despite policy changes intended to reduce regulatory barriers, buprenorphine prescribing has remained stable in the US between 2018 and 2022.^13^

The Michigan Opioid Collaborative (MOC) was founded in 2017 to comprehensively address barriers to providing evidence-based treatment for OUD, including supporting increased prescribing of buprenorphine.^14^ The MOC includes multiple components, including behavioral health consultants who proactively reach out to community clinics, webinars and trainings, and support from addiction experts. The present study sought to evaluate the association between the MOC program and buprenorphine prescribing in Michigan. To assess program outcomes, we used outcome data from the Michigan prescription drug monitoring program, which contains records of all buprenorphine prescriptions dispensed in the state. We compared counties that were receiving MOC services with counties not receiving services in their monthly number of unique prescribers and the number of unique patients receiving buprenorphine prescriptions.

## Methods

### Study Setting and Design

**Figure 1.**
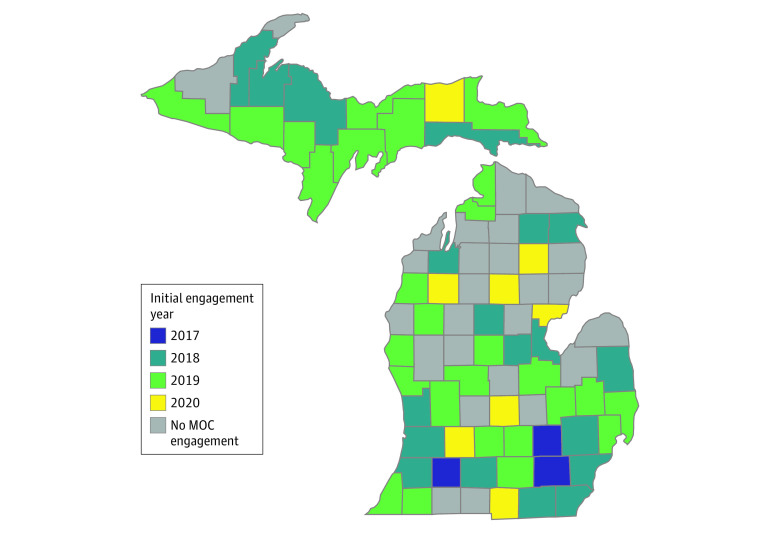
Status of Connection With Michigan Opioid Collaborative (MOC) for Counties in Michigan by Year The figure shows the calendar year when the first outreach occurred between the MOC program and the corresponding county in Michigan. Engagement was defined as any interactive communication between MOC and a practitioner, including email, phone, or in-person conversations.

This study is a county-level time-series evaluation of an outreach and education intervention to address barriers to MOUD prescribing. The program was expanded over time, with regions selected according to pragmatic considerations and priorities to address geographic gaps in treatment access, as identified by leaders at the Michigan Department of Health and Human Services (Figure 1). The project was in Michigan, a geographically large and diverse state in the Midwest, with a mix of urban, micropolitan, suburban, and rural counties. Data were included for all 83 counties in the state for the period of 2015 to 2020. The University of Michigan institutional review board evaluated the study and deemed it exempt from human participants’ review, and informed consent was not required because data were deidentified. This study followed the Strengthening the Reporting of Observational Studies in Epidemiology (STROBE) reporting guideline.

### The MOC Program

The MOC was created in a partnership between The Michigan Department of Health and Human Services and clinicians and other behavioral health experts at the University of Michigan and Michigan State University. The MOC aims to increase access to evidenced-based OUD care throughout Michigan by supporting clinicians, health care organizations, and communities. MOC offers a range of services, including same-day patient case consultations with addiction physicians; clinician and community education including trainings, webinars, and presentations; guidance for individual clinicians or clinics that wish to prescribe MOUD in a primary care setting; local community referrals for patient services including legal aid, housing assistance, food assistance, and specialty addiction treatment centers; clinician outreach to increase interest and acceptance of MOUD treatment; and supporting community coalitions.

The MOC has an interdisciplinary team including addiction physicians and researchers, behavioral health consultants (BHCs), communication experts, data analysts, program coordinators, and peer support specialists. BHCs are masters-level mental health clinicians regionally distributed to support all counties in Michigan. The local presence of the BHCs allows them to identify and respond to community needs.

### Data Sources and Measures

#### MOC Database

Staff document all contacts in a database, which was used to measure the key exposure variable for this study, MOC engagement. We defined an engagement as any bidirectional communication between MOC and a community practitioner, defined as any staff, including a counselor, nurse, medical assistant, peer support, physician, nurse practitioner, physician assistant, or others working in a clinical setting. Engagements encompassed several different bidirectional contacts, including a phone conversation, email exchange, or a BHC presentation. For example, this would include attending a community meeting to present on MOUD, a BHC reaching out to a clinic to ask if they are prescribing MOUD, or a clinic staff reaching out after a presentation to ask questions. Engagements could be initiated either on the practitioner side or by any MOC staff (BHCs, addiction physicians, or MOC administration). We did not include unidirectional activities such as BHCs attending a meeting (without presenting) or outreach attempts without response. We identified all instances of engagement occurring between October 2017 and August 2020. We classified each county on a per-month basis as to whether an engagement with a practitioner located in that county had occurred. After the first instance, that county was considered engaged henceforth. We deliberately opted for a low threshold to enhance the baseline similarity between counties with and without engagement.

#### Prescription Drug Monitoring Program

We obtained details of buprenorphine prescribing in Michigan from the Michigan Automated Prescription System (MAPS), the state’s prescription drug monitoring program.^15^ MAPS data are generated when controlled substances are dispensed by outpatient pharmacies throughout the state, as mandated by law. We used a deidentified version of the data that includes the medication formulation, date of fill, a prescriber identifier, and location of fill. Our prior comparison with other opioid dispensing data sources found this to be the most complete source available.^16^

### County Demographics

Demographic information for each county in Michigan were obtained from the 2019 Census Bureau’s Population Estimates Program (PEP)^17^ and the American Community Survey 5-year estimates.^18^ We incorporated self-identified race data into the study due to prior studies establishing race-based differences in buprenorphine treatment and access. We recategorized National Center for Health Statistics Urban-Rural Classification Scheme for Counties^19^ to (1) rural (noncore, micropolitan) and (2) metropolitan. County-level rates of uninsured individuals were taken from the Small Area Health Insurance Estimates,^20^ and rates of individuals in poverty (using the Census Bureau’s definition) were derived from the Small Area Income and Poverty Estimates.^21^ County-level prescription statistics in Michigan were calculated from MAPS.^15^

### Outcomes and Variable of Interest

The coprimary outcomes were the monthly density of active buprenorphine prescribers and patients receiving buprenorphine at the county level, which was defined as the number of physicians who prescribed buprenorphine per 100 000 population, and the number of patients who filled buprenorphine prescriptions per 100 000 population. The numerators were organized as panel data grouped by prescribers’ counties using MAPS records from May 1, 2015, to August 31, 2020. The denominator was derived from the 2019 PEP.^17^

The main variable of interest was the temporal association between MOC engagement and coprimary outcomes. For MOC-engaged counties, the engagement date (time 0) was defined as the month when the first engagement occurred between the MOC program and the corresponding county. For non–MOC-engaged counties, time 0 was set to be October 1, 2017, which was the established time of the MOC program. Notably, the selection of time 0 for non–MOC-engaged counties would not change the estimation of time trend in the linear regression model. Based on time 0, time since engagement, a linear time-trend variable was calculated as the number of months from time 0 to prescription date.

### Statistical Analysis

Difference-in-difference (DID) analyses were performed to evaluate the association between MOC engagement and outcomes. We compared overall changes in outcomes over time between MOC-engaged counties and nonengaged counties using linear fixed-effect models. The variable of interest was the interaction term of time since engagement and a binary indicator of postengagement period for MOC-engaged counties, after adjusting for time since engagement and the postengagement indicator. We also included county fixed effects in the model, which generates MOC treatment effects with only within-county associations, automatically controlling for any time-invariant state characteristics.^22^ The parallel trends for DID analysis required equivalent baseline temporal trends in active buprenorphine prescriber and patient densities between MOC-engaged and non–MOC-engaged counties in the absence of MOC engagement.^23^ To test the parallel trends assumption, we applied linear regression to data before the establishment of the MOC program. The model contained the fixed effect of county, time since engagement, and the interaction term between time since engagement and an indicator for MOC-engaged county, which is the main variable of interest (eTable 1 in Supplement 1). Model diagnostics indicated nonconstant variation, and thus, for all models, we used Huber-White SEs to derive heteroscedasticity-consistent inference.^24^ We further conducted a sensitivity analysis by implementing fixed-effect models on the data, with the exclusion of the initial three 2017-involved counties.

We also conducted county-specific analysis by applying linear regression models for each MOC-engaged county separately, except for Alger county, which had no buprenorphine prescriptions. The outcomes and variables of interest remained consistent with those previously mentioned in the linear fixed-effect models. Similarly, Huber-White SEs were used to accommodate nonconstant variation.^24^

All statistical analyses used R version 4.1.2 (R Project for Statistical Computing).^25^ A 2-sided *P* value less than .05 was considered statistically significant. Analyses were conducted from September 2021 to November 2023.

## Results

Michigan is made up of 83 counties with a total population size of 9 990 000. A total of 5 070 000 (50.8%) were female, 1 410 000 (14.1%) were African American or Black, 70 000 (0.7%) were American Indian and Alaska Native, 340 000 (3.4%) were Asian, 5 300 000 (5.3%) were Hispanic or Latino, 3000 (0.03%) were Native Hawaiian and Other Pacific Islander, and 7 470 000 (74.7%) were non-Hispanic White. The mean (SD) value of median age across counties was 44.8 (6.4). Among the 83 counties in Michigan, 57 (68.7%) built engagement with the MOC program between October 2017 and August 2020. After engaging 3 counties in 2017, the program expanded to practitioners in 19 counties (22.9%) in 2018 and 27 counties (32.5%) in 2019 across all urban or rural categories in the state and 8 more counties (9.6%) in 2020. The selection of the first 3 counties in 2017 was primarily driven by practical factors (ie, proximity to the University of Michigan). Subsequent counties engaged from 2018 to 2020 were strategically chosen to bridge geographical gaps in treatment access as identified by the Michigan Department of Health and Human Services and to achieve geographical distribution across the state. Figure 1 depicts the spatial distribution of MOC-engaged counties, and Table 1 describes the demographic and buprenorphine treatment characteristics for Michigan counties, stratified by year of engagement. Table 2 depicts the types and number of engagements over the study years. MOC engagements grew from 3 engagements in 2017 to 448 in 2020 (9.33 per county). Common topics included providing overview on the MOC programs (total of 141 engagements in 2020), providing information about the buprenorphine waiver process (102), discussing clinic workflow (84), general prescribing (35), and community resources (31).

**Table 1.** Characteristics of Counties in Michigan by Michigan Opioid Collaborative Engagement Year

Characteristic	Counties, mean (SD), %				
	2017 (n = 3)^a^	2018 (n = 19)	2019 (n = 27)	2020 (n = 8)^a^	Nonengaged (n = 26)
Demographics					
Population size in millions	2.75 (0.88)	2.31 (4.61)	1.38 (2.07)	0.34 (0.26)	0.3 (0.18)
Female	50.47 (0.60)	49.82 (1.72)	49.79 (1.75)	48.62 (3.36)	49.5 (1.18)
Male	49.53 (0.60)	50.18 (1.72)	50.21 (1.75)	51.38 (3.36)	50.5 (1.18)
Race and ethnicity					
African American or Black	8.27 (6.56)	5.14 (8.94)	6.1 (6.10)	2.24 (3.98)	1.28 (1.42)
American Indian and Alaska Native	0.47 (0.06)	2.32 (4.64)	2.39 (3.39)	1.44 (1.71)	1.03 (0.73)
Asian	4.4 (4.42)	1.64 (1.93)	1.26 (1.50)	0.59 (0.45)	0.56 (0.18)
Hispanic or Latino	4.23 (1.42)	4.45 (3.09)	4.24 (3.21)	2.48 (0.96)	3.18 (1.75)
Native Hawaiian and Other Pacific Islander	0.01 (<.01)	0.02 (0.04)	0.04 (0.05)	0.05 (0.05)	0.03 (0.05)
Non-Hispanic White	80.43 (12.31)	84.72 (11.80)	84.09 (8.47)	91.61 (6.08)	92.62 (2.83)
Age, mean (SD), y^b^	37.17 (5.50)	42.88 (5.55)	43.83 (6.14)	46.21 (5.73)	47.6 (6.31)
Urbanicity, No. (%)					
Rural	0	5 (6.02)	9 (10.84)	4 (4.82)	14 (16.87)
Micropolitan	0	6 (7.23)	5 (6.02)	2 (2.41)	12 (14.46)
Metropolitan	3 (3.61)	8 (9.64)	13 (15.66)	2 (2.41)	0
Uninsured, age <65 y	5.5 (0.72)	7.02 (1.71)	7.36 (1.37)	7.39 (1.38)	7.85 (0.93)
Unemployed	4.48 (0.92)	6.02 (2.00)	5.99 (1.46)	6.49 (2.39)	5.91 (1.34)
No high school or GED	5.3 (0.95)	9.25 (3.03)	9.13 (2.64)	10.6 (3.30)	9.75 (2.52)
Per capita income in past 12 mos, $	37908.33 (5165.07)	28836.53 (5360.75)	27943.26 (3744.18)	26833.12 (4185.65)	26921.46 (3668.54)
Poverty	10.23 (5.05)	12.69 (3.42)	13.81 (3.73)	13.8 (4.17)	13.1 (2.91)
Buprenorphine prescription in 2016					
Buprenorphine prescribers’ density in millions	26.78 (18.14)	14.43 (10.59)	9.26 (7.21)	6.32 (5.53)	9.01 (7.87)
Patients receiving buprenorphine density in millions	532.81 (512.19)	532.09 (553.63)	280.07 (356.12)	98.62 (164.55)	195.97 (296.82)

^a^ Data from October 2017 to August 2020 were used in the analysis.

^b^ Mean of the median age of counties was reported.

**Table 2.** Annual Counts for Michigan Opioid Collaborative (MOC) Engagements by Types

Engagement type	Engagements, No.^a^			
	2017 (n = 3)^b^	2018 (n = 189)	2019 (n = 505)	2020 (n = 448)^b^
MOC overview	1	100	263	141
Buprenorphine waiver process	0	4	55	102
Clinic workflow	1	41	75	84
General prescription	0	15	36	35
Community resources	1	4	36	31
Other topics	0	28	72	66

^a^ Multiple topics can be discussed in 1 engagement.

^b^ Data from October 2017 to August 2020 were used in the analysis.

There were no identified significant differences between MOC-engaged counties and non–MOC-engaged counties in temporal trends of density of active buprenorphine prescribers at baseline, with the estimated mean difference being −0.03 (95% CI, −0.1 to 0.03) prescribers per 100 000 population (*P* = .31) (eTable 1 in Supplement 1). There was also no significant difference between counties with engagement and without engagement before MOC engagement date in the rate of active patients receiving buprenorphine (mean difference, 0.49 [95% CI, −2.02 to 2.99] patients per 100 000 population; *P* = .70) (eTable 1 in Supplement 1). These results indicate that the parallel assumption holds.

**Figure 2.**
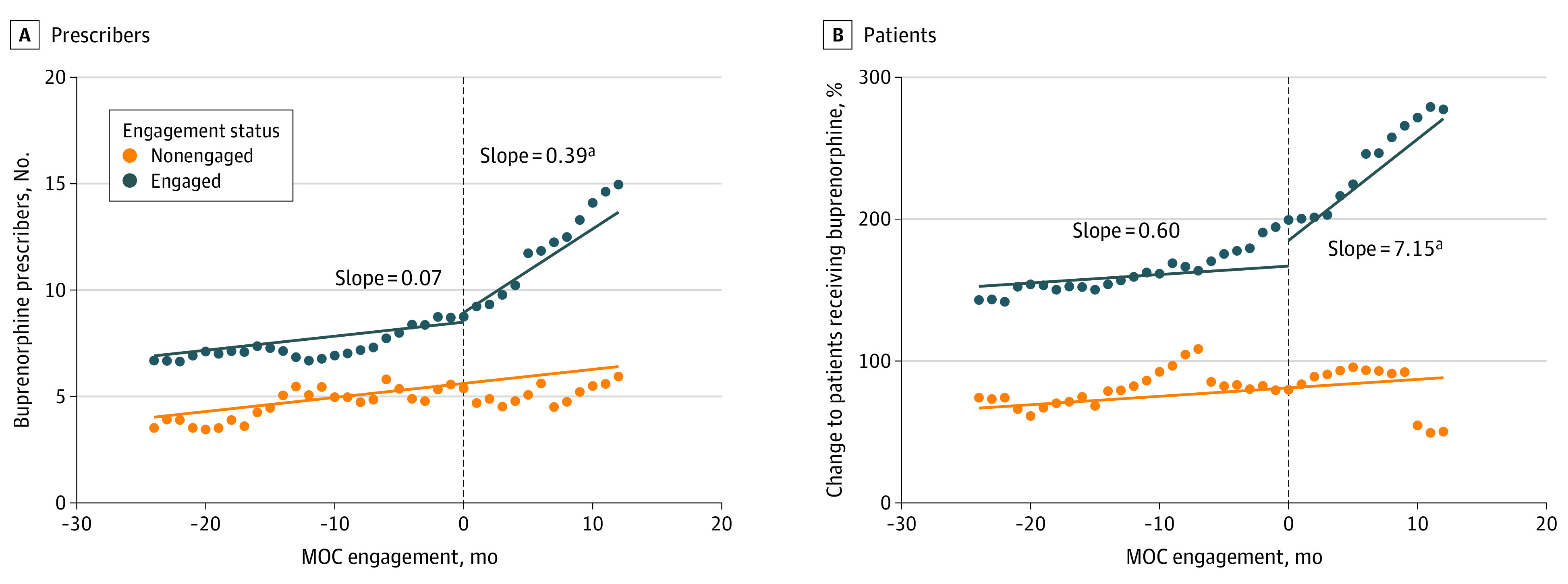
Overall Changes in Densities of Buprenorphine Prescribers and Patients Receiving Buprenorphine Before and After Michigan Opioid Collaborative (MOC) intervention The figure shows the association between MOC engagement with densities of A, active buprenorphine prescribers and B, patients receiving buprenorphine. Dashed vertical lines represent the month of MOC engagement. ^a^Change in slope (ie, rate of increase per month) from before to after the MOC engagement was statistically significant at *P* < .01.

Regarding the association between MOC engagement and buprenorphine prescription, we found a monthly increase of 0.07 (95% CI, 0.03 to 0.11; *P* = .001) buprenorphine prescribers per 100 000 population without MOC engagement, that is, the period before engagement with MOC in the case of MOC-engaged counties and throughout the entire follow-up period for non–MOC-engaged counties (Figure 2A). The average monthly increase in buprenorphine prescribers’ numbers per 100 000 for MOC-engaged counties during the postengagement period was 0.39, with the absolute difference being 0.33 (95% CI, 0.12 to 0.53) prescribers per 100 000 population (*P* = .002) compared with the prior engagement period (Figure 2A). The numbers of patients receiving buprenorphine increased by an average of 0.6 and 7.15 per 100 000 population per month in preengagement and postengagement periods, respectively, indicating an estimated additional 6.56 (95% CI, 2.09 to 11.02) patients receiving buprenorphine per 100 000 population (*P* = .004) monthly increase after engagement compared with before. We identified the same increasing pattern with respect to buprenorphine prescribers and patients receiving buprenorphine after excluding the first 3 engaged counties (eFigure in Supplement 1).

**Figure 3.**
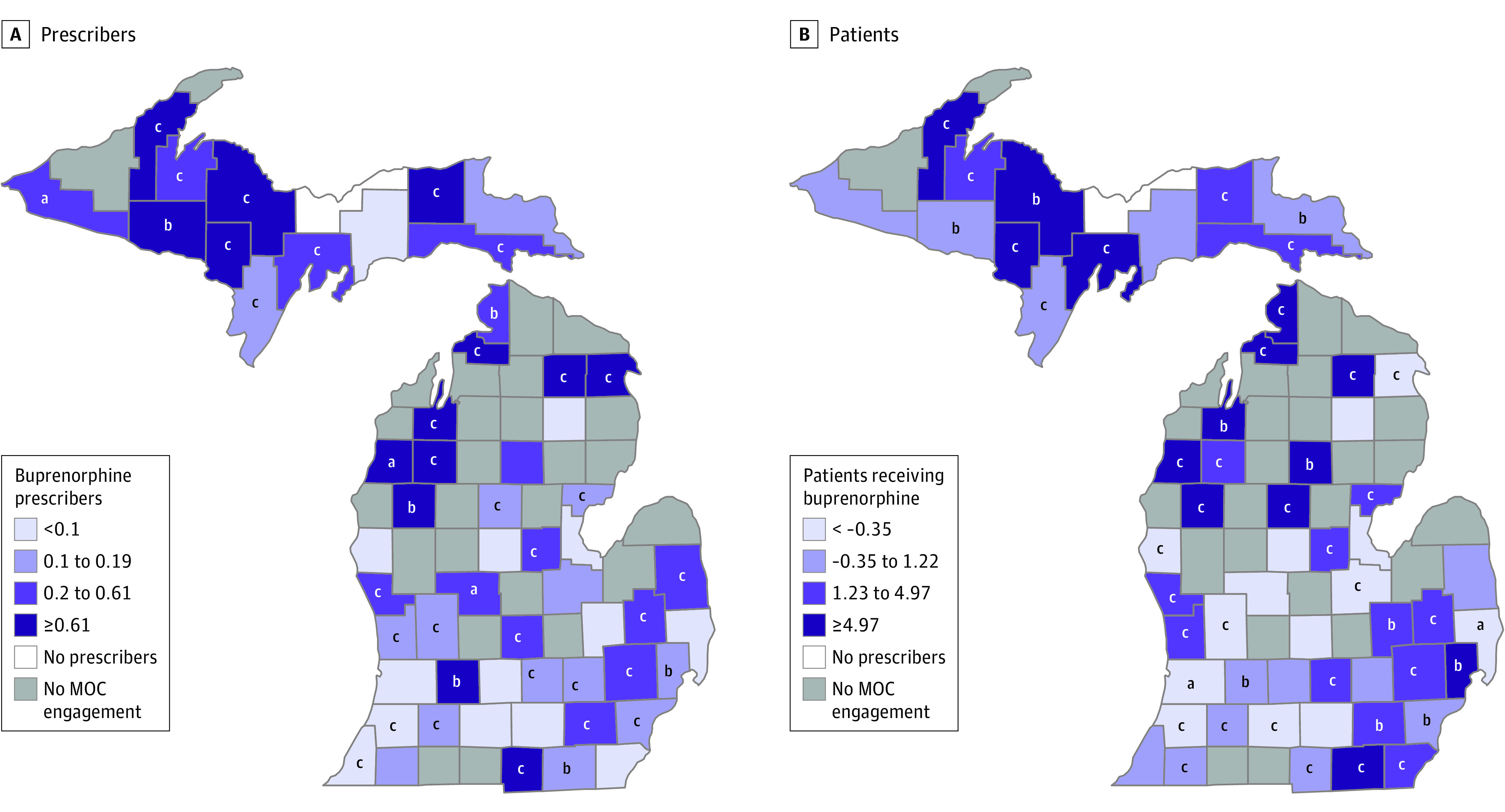
Association of Michigan Opioid Collaborative (MOC) Intervention With Density of Patients Receiving Buprenorphine and Prescribers for Each Michigan County The figure shows the association of MOC engagement with densities of A, active buprenorphine prescribers and B, patients receiving buprenorphine, along with the corresponding significance level for each MOC-engaged county, except for Alger county (white) which had no prescription records from May 1, 2015, to August 31, 2020. ^a^*P* < .05. ^b^*P* < .01. ^c^*P* < .001.

In county-specific analysis of the 56 MOC-engaged counties, engagement was associated with increased density of buprenorphine prescribers in 47 counties (statistically significant in 38). MOC engagement was associated with a decrease in prescribers’ density in 8 counties, and this decrease was significant in Van Buren (Figure 3A). We also identified a positive association between MOC engagement and density of patients receiving buprenorphine in 41 counties (statistically significant in 36). However, MOC engagement was found to be associated with a decrease in patients’ density in 15 counties, which was statistically significant in 8 counties (Figure 3B).

## Discussion

MOC was designed to meet the diverse needs of communities and improve access and quality of treatment for patients with OUD by using a multipronged approach of outreach, education, same-day consultation, and community engagement. The staggered implementation across counties allowed us to evaluate the outcomes of this statewide program using a DID approach. We found that, in counties engaged with MOC, the density of buprenorphine prescribers and patients filling prescriptions for buprenorphine increased more than in non–MOC-engaged counties.

In the county-specific analysis, we found that most counties experienced increases in buprenorphine prescribing, but a small number had significantly fewer prescribers or patients after MOC engagement. Potential reasons include (1) in counties with a small population size, the potential for 1 to 2 practitioners retiring or moving to have an outsized impact on density, and (2) the impact of changes are not capturable by the current data (eg, a new large clinic opens in a neighboring county).

Clinicians are often slow to take up new medical treatments, even when there is evidence establishing clear superiority over current practices.^26^ Patients with OUD should have access to MOUD given its proven ability to reduce mortality,^2^ but access is still woefully inadequate nationally.^27^ Many prior efforts to address this problem have targeted only 1 barrier in isolation, such as prior authorizations,^8^ clinician education,^12^ and eliminating education requirements to prescribe, the last of which was not associated with increased prescribing.^13^

MOC provided customized services that cater to different counties in Michigan. Although labor-intensive, this multipronged approach may be necessary to see meaningful increases in MOUD access. A basic principle of helping patients with substance use disorders is individualized care, known as meeting patients where they are. This principle can extend to communities, health care organizations, and clinicians. Despite tailoring to counties, outcome variability still exists, with a small number of engaged counties having decreases in access. This highlights the need to identify additional methods to overcoming barriers.

The success of the MOC highlights the potential of statewide collaboratives to meet the diverse needs of communities and improve access to evidence-based treatment for patients with OUD. The MOC was supported by contracts from the State of Michigan using funding received under the State Targeted Response (STR)^28^ and State Opioid Response opportunities by the Substance Abuse and Mental Health Services Administration. These initiatives resulted in several billions of dollars of funding to the states to support efforts to address the opioid overdose crisis. However, there has been limited evaluation of the effectiveness of these efforts.^29^ An Office of the Inspector General report on STR funding noted that the number of patients receiving MOUD was not tracked as part of the program.^28^ The present evaluation provides an example of how additional partnerships, in this case with the regulatory agency with control over prescription drug monitoring program data, are necessary to support quantitative evaluation. MOC receives ongoing support from Blue Cross Blue Shield of Michigan, but sustainability for this and similar programs is challenging in the context of federal funding to states to address the opioid crisis being allocated for 1 to 2 years at a time, often requiring new goals each funding cycle. Recent settlements between the states and opioid manufacturers and distributors may provide an opportunity for states to invest in expanding the clinician workforce. Future funding could prioritize the use of randomization, including timing of services as in a stepped wedge design,^30^ to better learn what community-level programs work best for addressing the opioid epidemic.

### Limitations

Our study has several limitations. MAPS data only contain filled prescriptions in Michigan, and some patients may have used pharmacies outside their county or outside the state. Because counties were not randomized, there is a possibility of selection bias and confounding. Communities and clinicians who were most open to prescribing buprenorphine may have been the participants most likely to engage with MOC. However, this is unlikely to explain the full association with engagement, as each county had many communities and practitioners, and the outcome measures were aggregate measures of buprenorphine prescribers and patients receiving buprenorphine. Further, the parallel trends analysis suggested that there were not preexisting differences in the trajectories of outcomes between those counties that did and did not receive MOC engagement.

## Conclusions

Buprenorphine is a life-sustaining medication for many patients with an OUD. Current access to buprenorphine is inadequate, and expanding access is key to addressing increasing rates of fatal overdoses. Patients benefit when they have access to local sources of substance use disorder care, but without education and support, many communities and practitioners are unable to overcome the many barriers that are often specific to their community and clinical settings. The MOC model presents 1 option to create a systematic approach to providing outreach and support but still meeting the unique needs of diverse communities. Payers and policy makers should expand low-cost resources such as educational collaboratives and remote delivery of addiction specialty support to increase access to evidence-based care, especially in rural and other underserved communities.

## Notes

Supplement 1. **eTable 1. **Parallel Assumption Validation by Applying Linear Regression to Data Before MOC Start Date **eTable 2. **Parallel Assumption Validation by Applying Linear Regression to Data Before MOC Start Date After Excluding the First 3 Engaged Counties **eFigure.** Overall Changes in Densities of Buprenorphine Prescribers and Patients Receiving Buprenorphine Before and After MOC Intervention With the Exclusion of the First 3 Engaged Counties

Supplement 2.
Data Sharing Statement
