# Supplementary material for: Prescriptions for Buprenorphine in Michigan Following an Education Intervention
Source: JAMA Netw Open. 2023 Dec 21;6(12):e2349103. doi: 10.1001/jamanetworkopen.2023.49103 (PMC10739087; doi:10.1001/jamanetworkopen.2023.49103)
Supplement: Supplement 1. — eTable 1. Parallel Assumption Validation by Applying Linear Regression to Data Before MOC Start Date eTable 2. Parallel Assumption Validation by Applying Linear Regression to Data Before MOC Start Date After Excluding the First 3 Engaged Counties eFigure. Overall Changes in Densities of Buprenorphine Prescribers and Patients Receiving Buprenorphine Before and After MOC Intervention With the Exclusion of the First 3 Engaged Counties [file jamanetwopen-e2349103-s001.pdf]

Supplemental Online Content

Chen L, Sethi S, Poland C, et al. Prescriptions for buprenorphine in Michigan following an education intervention. *JAMA Netw Open*. 2023;6(12):e2349103. doi:10.1001/jamanetworkopen.2023.49103

eTable 1. Parallel Assumption Validation by Applying Linear Regression to Data Before MOC Start Date

eTable 2. Parallel Assumption Validation by Applying Linear Regression to Data Before MOC Start Date After Excluding the First 3 Engaged Counties

eFigure. Overall Changes in Densities of Buprenorphine Prescribers and Patients Receiving Buprenorphine Before and After MOC Intervention With the Exclusion of the First 3 Engaged Counties

This supplemental material has been provided by the authors to give readers additional information about their work.

© 2023 Chen L et al. *JAMA Network Open.*

eTable 1. Parallel Assumption Validation by Applying Linear Regression to Data Before MOC Start Date

	Coefficient estimation (95% CI)	
	Number of Prescribers/100,000 Population	Number of Patients/100,000 Population
Time since engagement [c]	0.07 (0.02, 0.11) **	0.78 (-1.12, 2.68)
MOC engaged county * Time since engagement [c]	-0.03 (-0.1, 0.03)	0.49 (-2.02, 2.99)

a. *: P-value < 0.05; **: P-value < 0.01; ***: P-value < 0.001.
b. Linear models include controls for county fixed effects.
c. Engagement month was defined as the month when the first engagement occurred between the MOC program and the corresponding county for MOC-engaged counties. For non-MOC-engaged counties, engagement month was set to be Oct 1, 2017, which was the established time of the MOC program.

© 2023 Chen L et al. *JAMA Network Open.*

eTable 2. Parallel Assumption Validation by Applying Linear Regression to Data Before MOC Start Date After Excluding the First 3 Engaged Counties

	Coefficient estimation (95% CI)	
	Number of Prescribers/100,000 Population	Number of Patients/100,000 Population
Time since engagement [c]	0.07 (0.02, 0.11) **	0.78 (-1.12, 2.68)
MOC engaged county * Time since engagement [c]	-0.03 (-0.1, 0.03)	0.49 (-2.02, 2.99)

d. *: P-value < 0.05; **: P-value < 0.01; ***: P-value < 0.001.
e. Linear models include controls for county fixed effects.
f. Engagement month was defined as the month when the first engagement occurred between the MOC program and the corresponding county for MOC-engaged counties. For non-MOC-engaged counties, engagement month was set to be Oct 1, 2017, which was the established time of the MOC program.

Aiming to confirm if the observed differences were primarily shaped by these urban counties, we further conducted a sensitivity analysis by implementing fixed effect models on the data after exclusion of the initial three involved counties. Table S2 validated that the parallel assumption still held after excluding the first three engaged counties.

© 2023 Chen L et al. *JAMA Network Open.*

eFigure. Overall Changes in Densities of Buprenorphine Prescribers and Patients Receiving Buprenorphine Before and After MOC Intervention With the Exclusion of the First 3 Engaged Counties

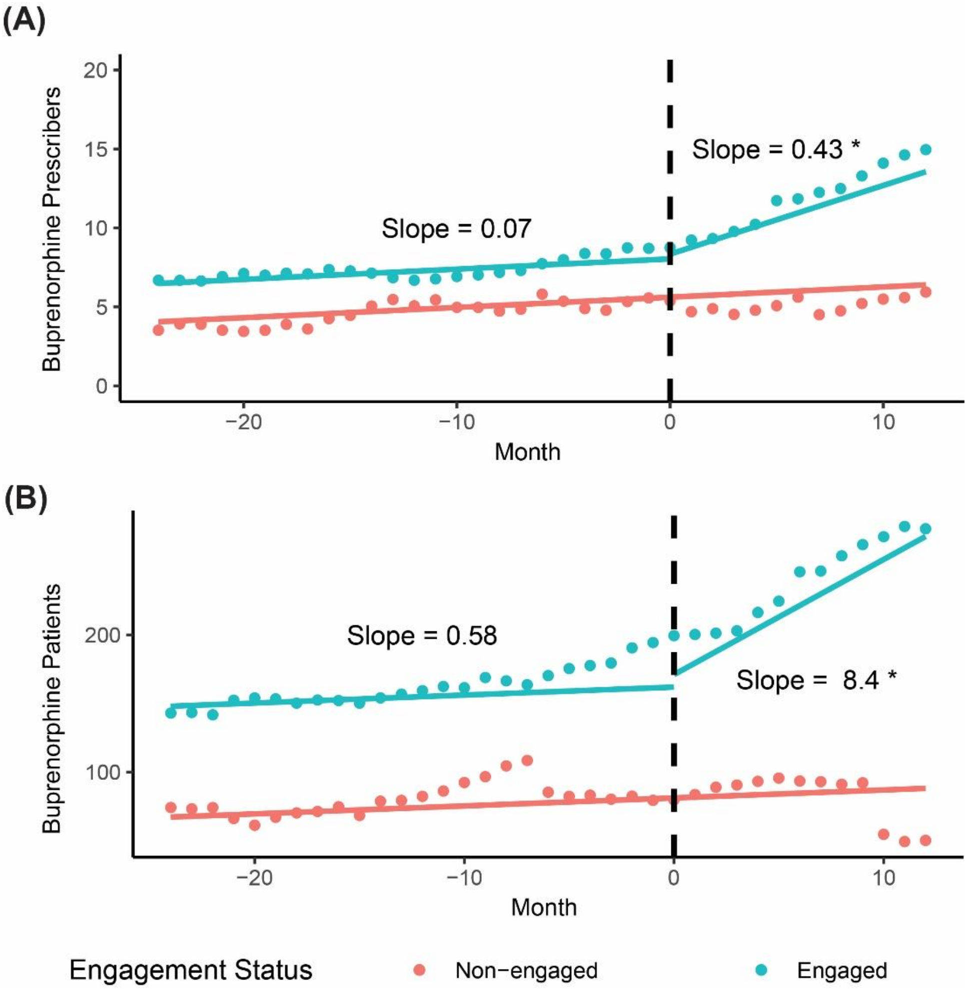

The figure shows the association between MOC engagement with densities of active Buprenorphine prescribers (A) and patients (B) with the exclusion of the first three engaged counties. Dashed vertical lines represent the month of MOC engagement.

* Change in slope (i.e., rate of increase per month) from before to after the MOC engagement was statistically significant at $P < 0.01$.

© 2023 Chen L et al. *JAMA Network Open.*
