# Supplementary material for: Prescriptions for Buprenorphine in Michigan Following an Education Intervention
Source: JAMA Netw Open. 2023 Dec 21;6(12):e2349103. doi: 10.1001/jamanetworkopen.2023.49103 (PMC10739087; doi:10.1001/jamanetworkopen.2023.49103)
Supplement: Supplement 2. — Data Sharing Statement [file jamanetwopen-e2349103-s002.pdf]

# Data Sharing Statement

Chen. Prescriptions for Buprenorphine in Michigan Following an Education Intervention. *JAMA Netw Open*. Published December 21, 2023. doi:10.1001/jamanetworkopen.2023.49103

**Data**
**Data available:** No

**Additional Information**
**Explanation for why data not available:** The use of data obtained from the Michigan Automated Prescription System (MAPS) is subject to certain regulations and procedures in order to protect the confidentiality of individuals. Access to MAPS data requires appropriate approval, and any output or analysis derived from the data must be submitted for review to ensure privacy compliance. To access MAPS data, researchers or individuals must adhere to the established protocols, which typically involve obtaining approval from the appropriate regulatory bodies or institutional review boards (IRBs). This approval ensures that the use of the data is in compliance with privacy regulations and safeguards.
